# microRNA-328 in exosomes derived from M2 macrophages exerts a promotive effect on the progression of pulmonary fibrosis *via* FAM13A in a rat model

**DOI:** 10.1038/s12276-019-0255-x

**Published:** 2019-06-04

**Authors:** Meng-Ying Yao, Wei-Hong Zhang, Wen-Tao Ma, Qiu-Hong Liu, Li-Hua Xing, Gao-Feng Zhao

**Affiliations:** 1 Department of Respiratory Intensive Care Unit, The First Affiliated Hospital of Zhengzhou University, Zhengzhou, 450052 PR China; 2 Department of Anatomy, Nursing College of Zhengzhou University, Zhengzhou, 450052 PR China; 3 Department of Thoracic Surgery, The First Affiliated Hospital of Zhengzhou University, Zhengzhou, 450052 PR China

**Keywords:** Cell biology, Molecular biology

## Abstract

Currently, exosome-enclosed microRNAs (miRs) in exhaled breath have potential for biomarker discovery in patients with pulmonary diseases. This study was performed to investigate the roles of M2 macrophage-derived exosomes expressing miR-328 in pulmonary fibrosis (PF). Microarray-based analysis was used to screen differentially expressed genes (DEGs) and regulatory miRs in PF. The miR-target relationship between FAM13A and miR-328 was confirmed. The expression of FAM13A and miR-328 was measured in PF rats, and gain- and loss-of-function assays were conducted to determine the regulatory effects of FAM13A and miR-328 on PF. In addition, exosomes derived from M2 macrophages were isolated and then cocultured with pulmonary interstitial fibroblasts to identify the role of these exosomes in PF. Furthermore, the effects of M2 macrophage-derived exosomes overexpressing miR-328 on pulmonary fibroblast proliferation and the progression of PF were assessed in vivo. miR-328 might perform a vital function in PF by regulating FAM13A. FAM13A expression was downregulated while miR-328 expression was upregulated in rats with PF, and a miR-target relationship between miR-328 and FAM13A was observed. Additionally, miR-328 overexpression and FAM13A silencing each were suggested to promote pulmonary interstitial fibroblast proliferation and the expression of Collagen 1A, Collagen 3A and α-SMA. Then, in vitro experiments demonstrated that M2 macrophage-derived exosomes overexpressing miR-328 contributed to enhanced pulmonary interstitial fibroblast proliferation and promoted PF. Furthermore, in vivo experiments confirmed the promotive effects of M2 macrophage-derived exosomes overexpressing miR-328 on the progression of PF. Collectively, the results showed that M2 macrophage-derived exosomes overexpressing miR-328 aggravate PF through the regulation of FAM13A.

## Lung disease: Small RNAs promoting pulmonary fibrosis

Studies in rats suggest that microRNAs, small molecules of ribonucleic acid, released by macrophage cells of the immune system can promote pulmonary fibrosis (PF), the formation of scar tissue in lungs. Gao-Feng Zhao, Li-Hua Xing and colleagues at The First Affiliated Hospital of Zhengzhou University in China investigated the role of microRNAs in rats with a form of PF that serves as a model for the disease in humans. Their findings confirm that specific microRNAs released in tiny membrane-bound sacs called exosomes interact with and inhibit a gene whose activity is known to be disrupted in PF. The protein encoded by this gene mediates crucial molecular signaling events in lung cells. Developing drugs that interfere with the activity of the microRNAs is a potential new treatment approach for PF.

## Introduction

Pulmonary fibrosis (PF) is a chronic lung-related disease with a high risk of death, in which idiopathic pulmonary fibrosis (IPF) is significantly severe^1,2^. It is believed that IPF is the type of interstitial pneumonia with the worst prognosis and a 50% 3–5-year mortality rate after diagnosis^3^. Ongoing worsening of dyspnea and lung dysfunction are notable features of IPF^4^. Although IPF pathogenesis remains to be elucidated, environmental exposures and occupational risk factors were suggested to be related to IPF in a recent study^5^. Since IPF and lung cancer share the same high susceptibility factors, they are considered to be closely related^6^. However, there is a lack of efficient treatments for IPF, and it is still a big challenge to manage IPF^7^.

Macrophages have been reported to play crucial roles in immunity, tissue turnover, organ development, and regeneration^8^. Misharin *et al*. found that macrophages are involved in promoting PF^9^. Macrophages can be divided into M1 and M2 activation phenotypes on the basis of function, and exosomes have been suggested to promote macrophage polarization^10^. Exosomes, which are smaller than 100 nm in diameter, originate from the endosomal compartment through the fusion of multivesicular bodies with the plasma membrane^11^. Exosomal-enclosed microRNAs (miRs) have been shown to improve tissue function and exert protective effects in vitro in various diseases and injuries^12^. For example, the exosomal miRs miR-4257 and miR-21 are upregulated in non-small cell lung cancer (NSCLC), and depleting miR-4257 and miR-21 can restrain the progression of NSCLC^13^. In addition, miR-328 has been suggested to play a positive role in accelerating the migration of NSCLC cells^14^. Abnormal miR-328 expression has also been found in some other cancers^15^. Moreover, miR-155 has been identified as a driving factor of fibrosis^16^. Family with sequence similarity 13, member A (FAM13A) is a susceptibility gene in chronic obstructive pulmonary disease (COPD) that has been implicated in COPD progression in genome-wide association studies^17^. Hobbs *et al*. indicated that FAM13A is closely associated with PF^18^. In addition, a previous report suggested that FAM13A polymorphisms affect the prognosis of patients with IPF^19^. Primer Premier 5.0 software analysis identified FAM13A as a target of M2 macrophage-derived exosomes carrying miR-328. Therefore, the present study was designed to explore the detailed mechanism of miR-328 in PF and its relationship with FAM13A.

## Materials and methods

### Ethics statement

All animal experiments were approved by the Ethics Committee of the First Affiliated Hospital of Zhengzhou University and followed the Guide for the Care and Use of Laboratory Animals. Best efforts were made to minimize the suffering of animals.

### Microarray analysis

The PF gene expression dataset GSE44723 from the Gene Expression Omnibus (GEO) database (https://www.ncbi.nlm.nih.gov/geo/), which consisted of gene expression data for fibroblasts from 7 patients with PF and 4 normal healthy people, was used to screen differentially expressed genes (DEGs). The gene annotation platform used was the GPL570 [HG-U133_Plus_2] Affymetrix Human Genome U133 Plus 2.0 Array. Normalization preprocessing of the gene expression data was performed using the affy package of R language (http://www.bioconductor.org/packages/release/bioc/html/affy.html), and DEGs were screened by the limma package (http://master.bioconductor.org/packages/release/bioc/html/limma.html) with a *p* value < 0.05 and |log fold change| > 2 as the threshold. A heat map of the DEGs was drawn by using the pheatmap package (https://cran.r-project.org/web/packages/pheatmap/index.html). Genes related to PF were identified in the DiGSeE database. Subsequently, the disease genes and DEGs correlated with PF were included in the String database (https://string-db.org/) to analyze gene interactions, and the established gene interaction network was visualized by Cytoscape 3.6.0 software^20^ to further screen the DEGs. miRs that might regulate the DEGs were predicted with miRWalk (http://mirwalk.umm.uni-heidelberg.de/).

### Establishment of PF rat models

A total of 80 specific pathogen-free (SPF) male Sprague-Dawley (SD) rats (weighing 233 ± 15 g, range: 210 g to 265 g) were provided by the Shanghai Institute of Laboratory Animals (Chinese Academy of Sciences, Shanghai, China). These animals were acclimatized at room temperature for 1 week with free access to food and water. In this study, the rat model with pulmonary interstitial fibrosis was established by intratracheal infusion of bleomycin (Nippon Chemical Co., Ltd., Tokyo, Japan, 1.5 mg/rat, dissolved in 0.3 mL of physiological saline). Among these rats, 78 were chosen for model establishment. Details about this method can be found in a previous publication^21^. The rats in the sham group were only injected with equal amounts of physiological saline.

### Cell culture

Macrophages: An SPF male SD rat weighing 250 g was anesthetized by intraperitoneal injection of 50 g/L sodium pentobarbital (Beijing Huayue Huanyu Chemical Co., Ltd., Beijing, Shanghai) at a dose of 30 mg/kg and then killed by exsanguination of the abdominal aorta. Bronchoalveolar lavage was performed with ice-cold phosphate-buffered saline (PBS). The bronchoalveolar lavage fluid was centrifuged at 402 × g for 10 min at 4 °C, and then the supernatant was removed, and the pellet was collected. Cell pellets were cultured in Dulbecco’s modified Eagle medium (DMEM) containing 10% fetal bovine serum (FBS) at a density of 1 × 10^9^ cells/L in an incubator with 5% CO_2_ and saturated humidity at 37 °C for 2 h. The cells were then resuspended in 4 mL of DMEM, harvested by detachment using 0.25% trypsin and inoculated into 6-well plates. M2 macrophages were induced by stimulation with interleukin-4 (IL-4; 10 ng/mL) for 24–96 h according to the method described by Odegaard et al.^22^.

Pulmonary interstitial fibroblasts: An SPF male SD rat weighing 250 g was anesthetized by intraperitoneal injection of 50 g/L pentobarbital sodium at a dose of 30 mg/kg and euthanized by exsanguination of the common carotid artery. Lung tissue (1 mm × 1 mm × 1 mm) was prepared and infiltrated with DMEM containing 10% FBS. Afterwards, tissue samples were evenly placed on one side of a 25 cm^2^ culture flask and placed in an incubator at 37 °C with 5% CO_2_ and 95% humidity. After 2 h of culture, when the small pieces of tissue had attached to the culture flask, 5 mL of DMEM containing 10% FBS was carefully added along the cell-free side of the culture flask. The flasks were allowed to incubate for another 96 h. After the cells were completely out of the tissue block, the block was gently removed with forceps. Subsequently, the medium was changed every 48 h, and the cells were subcultured when they were almost completely confluent.

### Determination of arginase 1 (ARG-1) activity

According to the method published by Lumeng et al.^23^, cells were lysed with 100 μL of 0.1% Triton X-100 after stimulation for 48 h, followed by the addition of 100 μL of 50 mmol/L Tris–HCl and 10 mmol/L MnCl_2_. After an incubation at 56 °C for 10 min, the cells were incubated with 100 μL of 0.5 mol/L arginine at 37 °C for 30 min, and then 800 μL of H_2_SO_4_/H_3_PO_4_ was added to terminate the reaction. Subsequently, the cells were incubated with 50 μL of 9% α-isonitrosopropiophenone at 95 °C for 30 min. The absorbance (D value) was measured at a wavelength of 540 nm, and a standard curve was established with urea.

### Flow cytometry

Anti-CD206 and anti-DECTIN-1 antibodies were used to assess stimulated cells by indirect immunofluorescence staining. A total of 5 × 10^5^ stimulated M2 macrophages were washed with PBS and resuspended in 100 μL of PBS, followed by incubation with 0.5 μg of unlabeled anti-CD206 antibody or 0.5 μg of unlabeled anti-DECTIN-1 antibody at 4 °C for 30 min. Then, the cells were washed with PBS twice to remove free antibody, resuspended in 100 μL of PBS, and incubated with 0.25 μg of phycoerythrin (PE)-labeled IgG (H + L) antibody at 4 °C for 30 min. After washing with PBS twice, the cells were resuspended in 0.3 mL of PBS and analyzed by flow cytometry. A PE-labeled IgG2b isotype antibody was used as a negative control (NC), and the M2 macrophages unable to bind with the antibody were considered a blank control.

### Isolation of exosomes

PEG6000 (16 g) and NaCl (5.18 g) were dissolved in 100 mL of ddH_2_O to prepare a 16% stock solution, which was then filtered by a 0.45 μm membrane for further use. Collected cell supernatant (200 mL) was centrifuged at 2000 × g for 10 min at 4 °C and at 10,000 × g for 30 min to remove cell debris and transferred to a new centrifuge tube. The collected supernatant was mixed with an equal volume of the PEG6000 stock solution at 4 °C overnight, followed by centrifugation at 10,000 × g for 60 min at 4 °C to remove the supernatant. Then, the precipitate was resuspended in PBS and centrifuged at 120,000 × g for 90 min at 4 °C. The collected precipitate contained exosomes. The exosomes were then resuspended in PBS and stored at -80 °C for subsequent experiments.

### Characterization of exosomes

Bio-transmission electron microscopy (Bio-TEM) was applied to observe the morphology of exosomes. A total of 20 μL of exosomes was added onto a copper mesh grid. After 1 min, the exosomes were dried with filter paper. The exosomes were then dried again with filter paper after the addition of 1 drop of 1% uranyl acetate for 1 min. After being placed under incandescent light, the exosomes were observed by TEM and imaged. The particle size of the exosomes was determined by dynamic light scattering (DLS). A total of 0.5 mL of exosomes was diluted with 4.5 mL of ultrapure water and passed through a 0.22 μm filter membrane with the size measured by a nanometer particle size analyzer. Later, the expression of the exosomal markers CD9, CD63 and CD81 was assessed by western blot analysis.

### Internalization of exosomes by pulmonary interstitial fibroblasts

PKH67 dye was diluted 1: 1000, completely mixed with 20 μg of exosome suspension, and placed at 37 °C for 15 min. The mixture was washed with PBS once at 100,000 × g for 70 min. Then, the PKH67-labeled exosomes were cocultured with pulmonary interstitial fibroblasts for 30 min. At 2 h and 24 h, confocal microscopy was used to observe the uptake of exosomes by pulmonary interstitial fibroblasts.

### Reverse transcription quantitative polymerase chain reaction (*RT-qPCR*)

Total RNA was extracted from tissue samples and cells using a Trizol kit (Invitrogen, Carlsbad, CA, USA). RNA was then reverse transcribed into cDNA according to the instructions of a TaqMan MicroRNA Assays Reverse Transcription primer (4427975, Applied Biosystems, Foster City, CA, USA). Real-time fluorescent quantitative PCR was performed using an ABI7500 quantitative PCR instrument (7500, Applied Biosystems). The reaction conditions were as follows: predenaturation at 95 °C for 10 min and 40 cycles of denaturation at 95 °C for 10 s, annealing at 60 °C for 20 s and extension at 72 °C for 34 s. U6 was used as the internal reference for miR-328, and glyceraldehyde-3-phosphate dehydrogenase (GAPDH) was used as the internal reference for FAM13A, Collagen 1 A, Collagen 3 A, and α-SMA. The primers for miR-328, FAM13A, Collagen 1 A, Collagen 3 A, andα-SMA were designed and synthesized by Wuhan Sangon Biotech Co., Ltd. (Wuhan, Hubei, China) (Table 1). The 2^-ΔΔCt^ method was employed to calculate the ratio of the relative expression of a target gene in the experimental group to that in the control group with the following formulas: ΔΔCt = ΔCt _experimental group_ - ΔCt _control group_ and ΔCt = Ct _target gene_ - Ct _internal reference_. Ct was the amplification cycle when the real-time fluorescence intensity reached the set threshold. Three independent experiments were conducted.

**Table 1:** Primer sequences for *RT-qPCR*

Gene	Primer sequence (5’-3’)
miR-328	F: ACCCCGTCCCCCCGTC
	R: ACAGACAGCATCACTCA
FAM13A	F: GATGGTGGACACACTCAGCA
	R: CGCCGCCTCATGAAAGAATG
Collagen 1 A	F: GCAATGCTGAATCGTCCCAC
	R: CAGCACAGGCCCTCAAAAAC
Collagen 3 A	F: TCACCACCACTGTCACGATG
	R: GTCACACAACGGAAGTTGGC
α-SMA	F: TGGCCACTGCTGCTTCCTCT
	R: GGGGCCAGCTTCGTCATACTCCT
U6	F: GCTTCGGCAGCACATATACTAAAAT
	R: CGCTTCACGAATTTGCGTGTCAT
GAPDH	F: TCCCTCAAGATTGTCAGCAATG
	R: AGATCCACAACGGATACATTGG

*RT-qPCR* reverse transcription quantitative polymerase chain reaction, miR-328, microRNA-328; FAM13A, family with sequence similarity 13, member A; α-SMA, α-smooth muscle actin; GAPDH, glyceraldehyde-3-phosphate dehydrogenase; F, forward; and R, reverse

### Western blot analysis

Total protein was extracted from cells, which were lysed by radioimmunoprecipitation assay (RIPA) Lysis Buffer (BB-3209, BestBio Co., Ltd., Shanghai, China), separated by sodium dodecyl sulfate-polyacrylamide gel electrophoresis (SDS–PAGE) and transferred to a polyvinylidene fluoride (PVDF) blotting membrane. After being blocked with a blocking solution for 1 h, the membrane was incubated with primary rabbit monoclonal antibodies, including anti-CD9 (1:2000, ab92726), anti-CD63 (1:1000, ab213090), anti-CD81 (1:1000, ab109201) and anti-FAM13A (1:500, ab122440) antibodies, overnight at 4 °C. All of the above antibodies were purchased from Abcam (Cambridge, MA, USA). The next day, the membrane was incubated with a horseradish peroxidase-labeled goat anti-rabbit immunoglobulin G (IgG) secondary antibody (1:1000, Wuhan Boster Biological Technology Co., Ltd., Wuhan, Hubei, China) for 1 h at 37 °C with shaking. The internal reference was GAPDH, and each experiment was repeated 3 times.

### Dual-luciferase reporter assay

Primer Premier 5.0 software was used to predict the binding sites for miR-328 in FAM13A. A dual-luciferase assay was performed to confirm whether FAM13A was a direct target of miR-328. The potential binding fragment for miR-328 in FAM13A and a mutant-type (MUT) FAM13A fragment in which the binding sites were mutated were cloned into separate PGLO vectors, namely, PGLO-FAM13A-wild type (WT) and PGLO-FAM13A MUT. The two reporter plasmids were separately cotransfected with miR-328 mimics or an NC plasmid into fibroblast-like synoviocytes. After transfection for 24 h, the cells were lysed and centrifuged at 25764 × *g* for 1 min, and the supernatant was collected. Luciferase activity was measured using the Dual-Luciferase® Reporter Assay System (E1910, Promega, Madison, WI, USA). The relative luciferase activity was calculated as the ratio of the activity of firefly luciferase to that of Renilla luciferase. The experiment was repeated 3 times.

### 5-Ethynyl-2′-deoxyuridine (EdU) labeling

Cells in the logarithmic growth phase were cultured in a 96-well plate (4 × 10^3^ cells per well). The cells in each well were incubated with 100 μL of 50 μM EdU for 2 h and then rinsed with PBS 1–2 times (5 min per time). Subsequently, the cells in each well were fixed with 50 μL of fixative solution (PBS containing 4% paraformaldehyde) for 30 min at room temperature, incubated with 50 μL of 2 mg/mL glycine for 5 min on a swing bed, penetrated with 100 μL of penetrant (PBS containing 0.5% Triton X-100) for 10 min, and washed with PBS for 5 min. After that, the cells in each well were incubated with 100 μL of 1 × Apollo^®^ staining reaction solution on a swing bed at room temperature for 30 min in the dark. Subsequently, cells were rinsed with 100 μL of penetrant (PBS containing 0.5% Triton X-100) 2–3 times (10 min per time) on a swing bed and washed with 100 μL of methanol 1–2 times (5 min per time) and with PBS for 5 min. Reagent F was diluted with deionized water at a ratio of 1:100 to prepare a 1 × Hoechst33342 reaction solution, which was then added to the cells in each well and incubated on a swing bed for 30 min in the dark, followed by washing with 100 μL of PBS 1–3 times.

### Immunohistochemistry (IHC)

IHC was performed on 4% buffered formalin-fixed and paraffin-embedded sections (4 μm thick). Next, the sections were incubated with 100 μL of primary antibodies, including rabbit-anti α-SMA (1:500, ab108424, Abcam, Cambridge, MA, USA) and anti-Collagen I (5 μg/mL, ab34710, Abcam, Cambridge, MA, USA) antibodies. Then, the sections were incubated with a biotinylated goat anti-rabbit secondary antibody (1:100, HY90046, Shanghai Hengyuan Biotechnology Co., Ltd., Shanghai, China).

### Masson staining

Paraffin-embedded sections of lung tissue were dewaxed and hydrated, followed by chromation treatment or mercury salt removal. The nucleus was stained with Regaud hematoxylin dye for 5–10 min and Masson Ponceau Red Acid Fuchsin Solution for 5–10 min. Then, the sections were developed with 1% phosphomolybdic acid aqueous solution for 3–5 min and stained with an aniline blue or a light green dye solution for 5 min.

### Cell treatment

A total of 400 μL of serum-free DMEM, 1.5 μg of core plasmid, 1.5 μg of viral packaging plasmid, and 6 μL of TurboFect (TurboFect:plasmid = 2:1) were mixed in a tube and allowed to stand for 15–20 min. The mixture was added dropwise into adherent cells, which were then incubated in a 5% CO_2_ incubator at 37 °C for 6–10 h. After the removal of the medium, the cells were incubated with 2.5 mL of complete medium. After ~40 h, the supernatant containing lentivirus was collected for infection or aliquoted and stored at -80 °C for subsequent experiments. The cells were then plated in 6-well plates or 12-well plates until the cell confluence reached 60%. Then, the cells were incubated with 5 μg/mL polybrene in a 37 °C incubator.

### In vivo experiment

A total of 14 d after PF model establishment, 42 rats in the PF group were injected with 100 μg of lentiviral-packaged plasmid, miR antagomir and exosomes *via* the tail vein. The rats in the control group were subcutaneously injected with an equal amount of PBS. After 2 weeks, the rats were anesthetized by intraperitoneal injection of 2% phenobarbital, and lung tissue samples were collected for Masson staining and IHC staining.

### Statistical analysis

Statistical analysis was performed with SPSS 21.0 software (IBM Corp., Armonk, NY, USA). Measurement data are expressed as the mean ± standard deviation. Data that were normal with homogenous variance were compared between two groups by an unpaired *t* test, which was used for all comparisons between two groups. Among multiple groups, data were analyzed with one-way analysis of variance (ANOVA) or repeated measurement ANOVA. Data with a skewed distribution or variance heterogeneity were analyzed by a rank-sum test. *p* < 0.05 indicated statistical significance.

## Results

### rno-miR-328(a-3p) may be related to PF *via* FAM13A

**Fig. 1:**
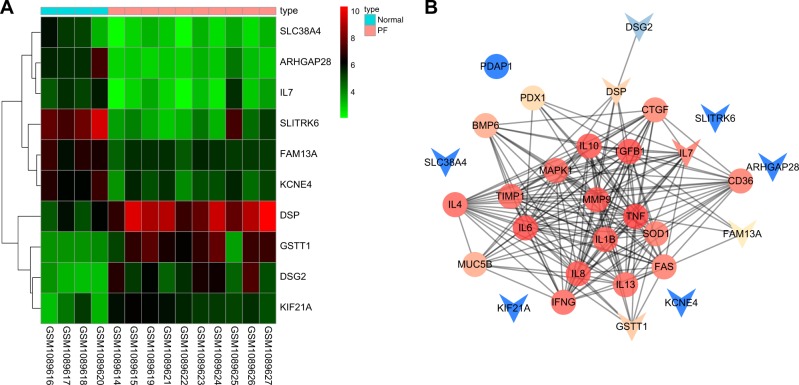
**FAM13A and miR-328 may work in tandem to affect PF**. **a** Heat maps of the top 10 DEGs in the PF gene expression dataset GSE44723. The abscissa suggested the sample number, the ordinate indicated the DEGs, and the right upper histogram indicated the color gradation. Each rectangle in the panel corresponded to one sample expression value. **b** The interaction network of DEGs and disease genes related to PF, where arrows indicate DEGs, and circles indicate disease genes. miR-328 microRNA-328, FAM13A family with sequence similarity 13, member A, PF, pulmonary fibrosis, and DEGs, differentially expressed genes

First, R language was used for a differential analysis of the microarray data in GSE44723 for screening DEGs related to PF. Heat maps of the top 10 DEGs in GSE44723 (ARHGAP28, FAM13A, SLITRK6, DSP, DSG2, SLC38A4, KIF21 A, KCNE4, IL7, and GSTT1) are shown in Fig. 1a and were subsequently analyzed. PF-related disease genes were identified in DiGSeE, and the top 20 genes (TNF, IL1B, TGFB1, IL8, BMP6, PDX1, IFNG, MUC5B, PDAP1, CD36, IL4, IL6, IL10, TIMP1, MAPK1, SOD1, IL13, CTGF, MMP9 and FAS) were selected as disease genes. The disease genes and DEGs were included in the String database for interaction analysis, and the interaction network of the DEGs and disease genes related to PF (Fig. 1b) was established. FAM13A, DSP, IL7, and GSTT1 were the DEGs most correlated with PF. As shown in the DEG heat maps (Fig. 1a), FAM13A and IL7 were poorly expressed in PF. IL7 is an antifibrosis factor^24,25^, and a FAM13A gene polymorphism is thought to be associated with PF;^19^ therefore, we investigated the role of FAM13A in PF.

miRs that might regulate FAM13A were predicted with miRWalk, and the top 20 miRs are shown in Table 2. The TarPmiR algorithm was utilized to predict the binding sites for miRs in the DEGs in the miRWalk database^26^. Binding probability and folding energy are parameters of TarPmiR. We noted that rno-miR-328a-5p and rno-miR-328a-3p were the miRs with lower folding energy that exhibited larger binding probabilities, indicating that they were more likely to target FAM13A. Through searching the miRBase database (http://www.mirbase.org/), the previous IDs of rno-miR-328a-5p and rno-miR-328a-3p were identified as rno-miR-328a* and rno-miR-328(a), respectively. Previous studies have suggested that miR-328 promotes myocardial fibrosis^27,28^, but its effect on PF is unclear. Therefore, we assumed that rno-miR-328(a-3p) might affect PF by targeting FAM13A.

**Table 2:** Top 20 miRs regulating FAM13A

miRNAID	Start	End	Binding probability	Energy	Number of pairings	Binding region length	Longest consecutive pairings
rno-miR-322–5p	2219	2245	0.846	−20.1	19	26	7
rno-miR-322–3p	597	613	0.846	−18.6	14	16	8
rno-miR-323–5p	1615	1628	0.923	−22.2	11	13	8
rno-miR-323–3p	2207	2250	1	−17.1	13	15	13
rno-miR-301a-5p	752	778	0.923	−19	16	21	9
rno-miR-324–5p	1453	1514	0.815	−18.1	20	61	12
rno-miR-324–3p	2286	2305	1	−25.2	16	19	12
rno-miR-325–5p	944	988	1	−24	19	35	12
rno-miR-325–3p	2152	2200	1	−22.8	11	13	11
rno-miR-326–5p	1687	1719	1	−24.3	13	19	8
rno-miR-326–3p	2101	2122	1	−19.9	16	21	7
rno-miR-327	999	1020	1	−21.3	16	21	9
rno-let-7d-5p	2298	2334	1	−21.3	14	18	8
rno-let-7d-3p	388	437	0.923	−22.8	18	49	11
rno-miR-328a-5p	988	1017	1	−31.2	17	21	9
rno-miR-328a-3p	12	69	1	−24.9	19	25	14
rno-miR-329–5p	102	127	0.884	−24.3	20	25	11
rno-miR-329–3p	2236	2250	0.923	−16.5	11	14	6
rno-miR-330–5p	1965	1982	1	−24.6	15	17	8
rno-miR-330–3p	1581	1603	0.923	−22.5	15	22	7

*miR/miRNA* microRNA; and FAM13A, family with sequence similarity 13, member A

### miR-328 expression is upregulated in a rat model of PF

Next, we investigated the altered expression of miR-328 in the rat model of PF by *RT-qPCR*. No rats died during the modeling process in either the sham or PF group so the survival rate was 100%. We compared the lung weight, lung coefficient, hydroxyproline (HYP) content, alveolar inflammation, and degree of PF between the sham and PF groups. The results are shown in Table 3. Compared with those in the lungs of the rats in the sham group, the lung weight, lung coefficient, and HYP content in the lungs of the rats in the PF group significantly increased with evident alveolar inflammation 3 d after modeling. The degree of PF was significantly higher in the PF group than in the sham group 28 d after modeling.

**Table 3:** PF-related parameters in rats after modeling

Group		Lung weight (g)	Lung coefficient (g/g)	HYP content (mg/g)	Alveolar inflammation	PF degree
Sham group		1.39 ± 0.14	0.31 ± 0.03	1.18 ± 0.12	1.04 ± 0.11	1.28 ± 0.12
	3 d	2.54 ± 0.21*	0.97 ± 0.1*	1.68 ± 0.15*	2.02 ± 0.18*	2.36 ± 0.16*
PF group	14 d	2.49 ± 0.24*	1.22 ± 0.11*	2.29 ± 0.23*	3.11 ± 0.28*	2.59 ± 0.26*
	28 d	2.59 ± 0.23*	0.86 ± 0.09*	2.64 ± 0.21*	2.94 ± 0.30*	3.16 ± 0.31*

*PF* pulmonary fibrosis, and *HYP* hydroxyproline. All results were measurement data and were analyzed using one-way analysis of variance. The results are expressed as the mean ± standard deviation; * *p* *<* 0.05, *vs*. the sham group; the sham group, *n* = 18; and the model group, *n* = 18

**Fig. 2:**
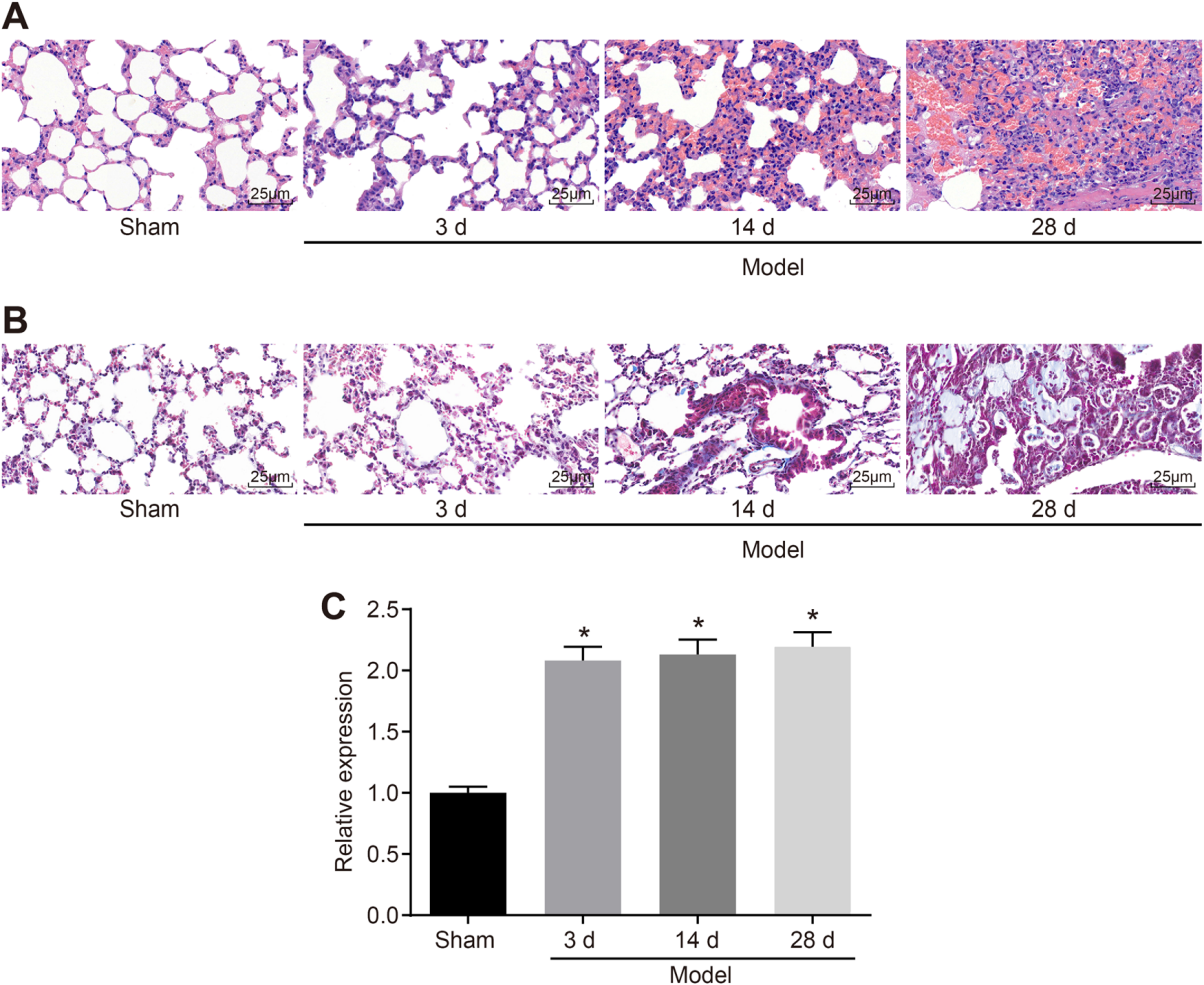
**miR-328 expression is upregulated in PF**. **a** HE staining of lung tissue 3 d, 14 d and 28 d after modeling (400 × ). **b** Masson staining of lung tissue 3 d, 14 d and 28 d after modeling (400 × ). **c**, miR-328 levels 3 d, 14 d and 28 d after modeling examined by *RT-qPCR*. * *p* *<* 0.05 *vs*. the sham group; miR-328, microRNA-328; HE, hematoxylin-eosin; *RT-qPCR*, reverse transcription quantitative polymerase chain reaction; and PF, pulmonary fibrosis. All results were measurement data and were analyzed using one-way analysis of variance. The results are expressed as the mean ± standard deviation; the sham group, *n* = 18; and the PF group, *n* = 18

HE staining and Masson staining showed that (Fig. 2a, b) in the sham group, no obvious lesions were observed in the lung tissue, and no inflammatory cell infiltration or pulmonary collagen deposition was detected. However, inflammatory cell exudation in some areas and widened alveolar spaces were observed 3 d after model establishment in the rats of the PF group. Fourteen days after modeling, obvious thickening of the alveolar space and inflammatory cell infiltration were found in the PF group by HE staining. In addition, significantly more fibroblasts, collagen fibers and fibrous tissue hyperplasia and fewer alveolar structures were detected in the PF group by Masson staining. On the 28th day after modeling, HE staining suggested the destruction of alveolar structure, proliferation of fibroblasts, and formation of scar-like fibrous tissue in patchy distributions, but peripheral lung parenchymal inflammation was decreased. Masson staining also showed notably increased amounts of blue collagen fibers.

In addition, miR-328 expression in the sham and PF groups 28 d after modeling was assessed by *RT-qPCR* (Fig. 2c), which revealed that compared with that in the sham group, the expression level of miR-328 in the PF group was significantly higher (*p* *<* 0.05); therefore, miR-328 expression was dysregulated in PF.

### miR-328 targets FAM13 A

**Fig. 3:**
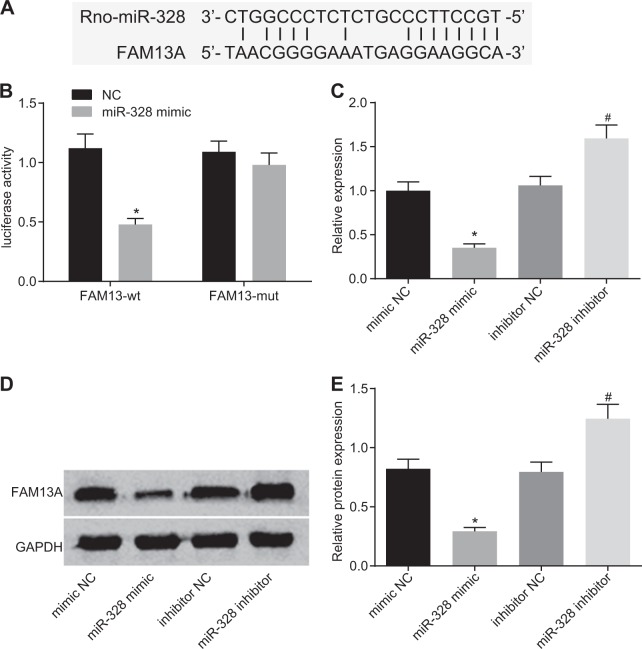
**miR-328 negatively targets FAM13A**. **a** The potential binding sites for the interaction between miR-328 and FAM13A. **b** The luciferase activity of FAM13A-WT and FAM13A-MUT with or without the cotransfection of miR-328. * *p* *<* 0.05 *vs*. the NC group. **c** The mRNA level of FAM13A after altering miR-328 activity. **d**, **e** The protein level of FAM13A after altering miR-328 expression. *p *<* 0.05 *vs*. the mimic NC group; ^#^*p* *<* 0.05 *vs*. the inhibitor NC group. miR-328, microRNA-328; FAM13A, family with sequence similarity 13, member A; *RT-qPCR*, reverse transcription quantitative polymerase chain reaction; NC, negative control; WT, wild type; and MUT, mutant type. The results were measurement data and are expressed as the mean ± standard deviation. The data in Fig. 3b were analyzed using an unpaired *t*-test, and the data in Fig. 3c and e were analyzed using one-way analysis of variance; the experiment was conducted in triplicate

The online website miRWalk predicted that miR-328 might negatively regulate FAM13A, and the potential binding site for miR-328 in FAM13A was identified by Primer Premier 5.0 (Fig. 3a). Subsequently, a dual-luciferase assay was performed to confirm the relationship between miR-328 and FAM13A. The luciferase activity of FAM13A WT in the miR-328 mimic group exhibited a notable reduction compared with that in the NC group (*p* *<* 0.05), while no significant difference was found in the luciferase activity of FAM13A MUT between the miR-328 mimic group and the NC group (*p* *>* 0.05) (Fig. 3b). To further confirm the miR-target relationship between miR-328 and FAM13A, we determined the mRNA and protein levels of FAM13A in the mimic NC, miR-328 mimic, inhibitor NC and miR-328 inhibitor groups by *RT-qPCR* and western blot analysis (Fig. 3c–e). Compared with that in the mimic NC group, the mRNA and protein expression of FAM13A in the miR-328 mimic group was markedly decreased. The expression of FAM13A was significantly increased in the miR-328 inhibitor group compared with the inhibitor NC group (*p* *<* 0.05). Based on the results above, we concluded that miR-328 could target FAM13A and downregulate its expression.

### Inhibition of miR-328 suppresses the proliferation of pulmonary interstitial fibroblasts in vitro by upregulating FAM13A expression

**Fig. 4:**
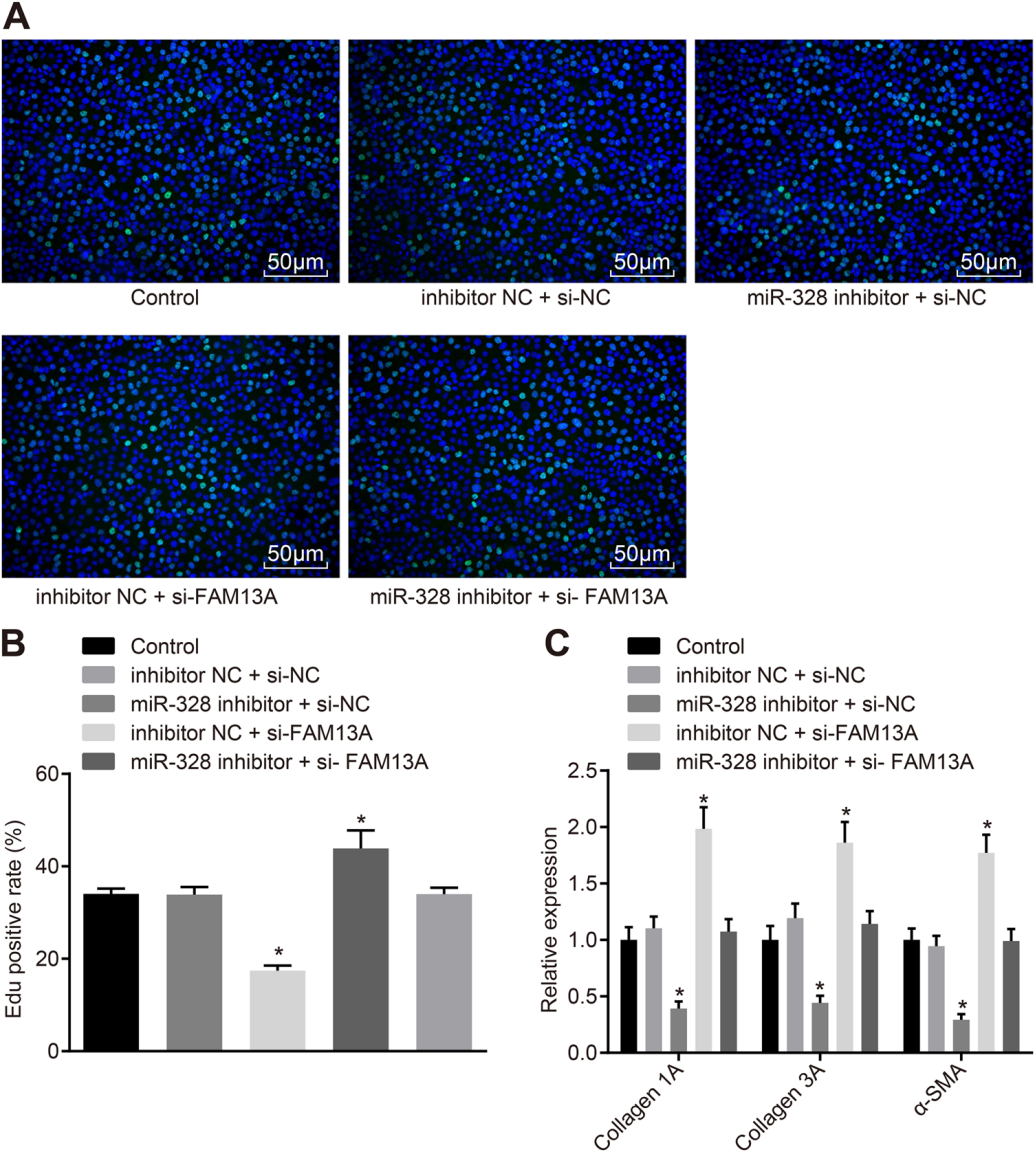
**miR-328 inhibition suppresses the proliferation of pulmonary interstitial fibroblasts in vitro by promoting FAM13 expression**. **a** EdU labeling to analyze the proliferation of pulmonary interstitial fibroblasts after the inhibition of miR-328 and silencing of FAM13 (200 × ). **b** Quantification of the results in A; C, *RT-qPCR* to examine the expression of Collagen 1A, Collagen 3A and α-SMA after the inhibition of miR-328 or silencing of FAM13. miR-328, microRNA-328; FAM13A, family with sequence similarity 13, member A; EdU, 5-ethynyl-2’-deoxyuridine; α-SMA, α-smooth muscle actin; *RT-qPCR*, reverse transcription quantitative polymerase chain reaction; and NC, negative control. *p *<* 0.05 *vs*. the inhibitor NC + si-NC group. The results were measurement data and were analyzed using one-way analysis of variance. The results are expressed as the mean ± standard deviation. The experiment was conducted in triplicate

The effects of miR-328 or FAM13A on the proliferation and function of pulmonary interstitial fibroblasts were then evaluated. The miR-328 inhibitor or si-FAM13A was transfected into pulmonary interstitial fibroblasts, and proliferation was detected by EdU (Fig. 4a, b). The mRNA levels of Collagen 1A, Collagen 3A and α-SMA were determined by *RT-qPCR* (Fig. 4c). Compared with the inhibitor NC + si-NC group, the miR-328 inhibitor + si-NC group showed a decline in the proliferation rate of pulmonary interstitial fibroblasts and decreases in the expression levels of Collagen 1A, Collagen 3A, and α-SMA, while the inhibitor NC + si-FAM13A group exhibited the opposite results (*p* *<* 0.05). Therefore, the inhibition of miR-328 could upregulate the expression of its target gene FAM13A; inhibit the expression of Collagen 1A, Collagen 3A, and α-SMA in pulmonary interstitial fibroblasts; and suppress the proliferation of pulmonary interstitial fibroblasts.

### M2 macrophages promote pulmonary interstitial fibroblast proliferation by secreting exosomes

**Fig. 5:**
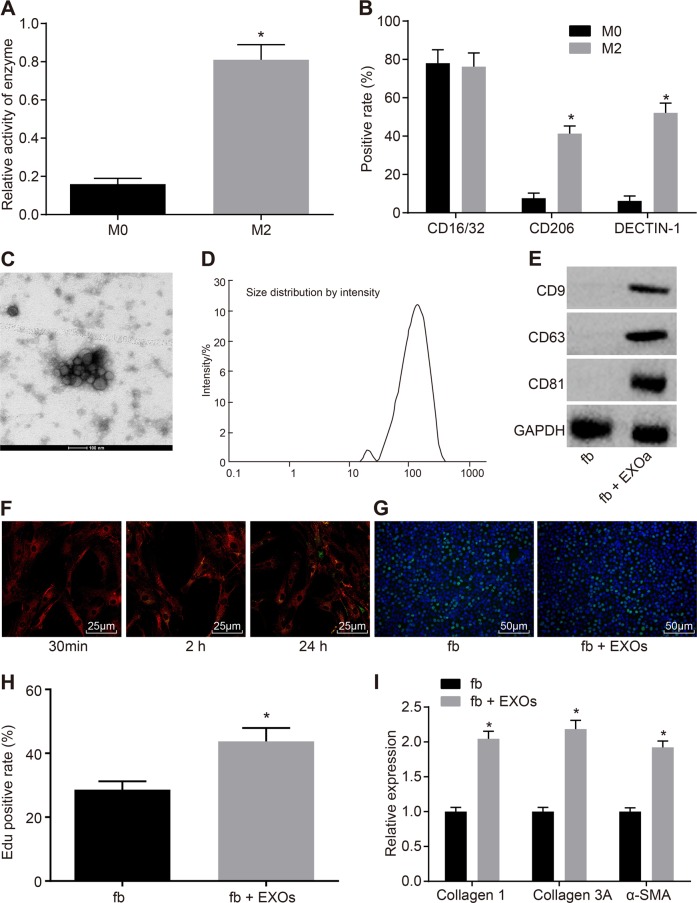
**M2 macrophage-secreted exosomes promote pulmonary interstitial fibroblast proliferation**. **a** Arg-1 activity of M0 and M2 macrophages, *p *<* 0.05 *vs*. M0 macrophages. **b** Positive expression rates of CD9, CD63 and CD81 in M0 and M2 macrophages, * *p* *<* 0.05 *vs*. M0 macrophages. **c** Exosome morphology observed by TEM. D, Exosome particle size analysis. **e** The protein levels of the exosomal markers CD9, CD63 and CD81 examined by western blot analysis. **f** The internalization of M2 macrophage-secreted exosomes by pulmonary interstitial fibroblasts at different time points; green indicates PKH67-labeled exosomes, and red represents pulmonary interstitial fibroblasts, bar = 25 μm. **g**, Edu labeling to examine the proliferation of pulmonary interstitial fibroblasts after a coculture with exosomes (200 × ). H, Quantification of the results in G. * *p* *<* 0.05 *vs*. pulmonary interstitial fibroblasts. I, Expression of Collagen 1 A, Collagen 3 A and α-SMA in pulmonary interstitial fibroblasts after a coculture with exosomes, as measured by *RT-qPCR*. * *p* *<* 0.05 *vs*. pulmonary interstitial fibroblasts. Arg-1, arginase 1; EdU, 5-ethynyl-2’-deoxyuridine; α-SMA, α-smooth muscle actin; and *RT-qPCR*, reverse transcription quantitative polymerase chain reaction. The results were measurement data and were analyzed using an unpaired *t* test. The results are expressed as the mean ± standard deviation. The experiment was conducted in triplicate

Next, we examined the effect of M2 macrophage-released exosomes on the genesis of interstitial fibroblasts. Previous studies have suggested a positive role for M2 macrophages in PF^29,30^, so we intended to investigate whether M2 macrophages alter the development of PF through the secretion of exosomes. M2 macrophages were induced in vitro by IL-4. The Arg-1 activity of the M2 macrophages was significantly increased compared with that of unstimulated M0 macrophages (*p* *<* 0.05) (Fig. 5a). There was no significant difference in the positive rate of CD16/32 expression between the M0 and M2 macrophages (*p* *>* 0.05), while the positive expression rates of CD206 and DECTIN-1 were significantly higher in the M2 macrophages than in the M0 macrophages (*p* *<* 0.05) (Fig. 5b). Exosomes were isolated from M2 macrophages and observed by electron microscopy (Fig. 5c). Additionally, the particle size of the exosomes was measured by DLS (Fig. 5d), and the levels of the exosomal markers CD9, CD63, and CD81 were determined by western blot analysis (Fig. 5e) to confirm the successful isolation of exosomes. Confocal microscopy was used to observe the uptake of exosomes by pulmonary interstitial fibroblasts. The results showed that after PKH67-labeled exosomes were cocultured with pulmonary interstitial fibroblasts for 30 min, the slight green fluorescence of the PKH67-labeled exosomes could be observed among pulmonary interstitial fibroblasts, which indicated that a small number of the PKH67-labeled exosomes entered the pulmonary interstitial fibroblasts. After 2 h of coculture, green fluorescence could be observed in a small amount of the pulmonary interstitial fibroblasts and was mainly found in the cytoplasm, which revealed that the PKH67-exosomes mainly existed in the cytoplasm after being taken up by the pulmonary interstitial fibroblasts. When the coculture time was extended, an increasing number of the pulmonary interstitial fibroblasts showed green fluorescence, indicating an increased number of the PKH67-labeled exosomes were taken up by the pulmonary interstitial fibroblasts. At 24 h, the uptake of the PKH67-labeled exosomes by the pulmonary interstitial fibroblasts was very obvious, which indicated that pulmonary interstitial fibroblasts could internalize exosomes secreted by M2 macrophages (Fig. 5f). To investigate the effects of exosomes on the proliferation of pulmonary interstitial fibroblasts, we performed EdU labeling (Fig. 5g, h) and *RT-qPCR* with pulmonary interstitial fibroblasts and pulmonary interstitial fibroblasts cocultured with exosomes (Fig. 5i). Compared with that of the pulmonary interstitial fibroblasts alone, the proliferation rate of the pulmonary interstitial fibroblasts cocultured with exosomes was significantly increased, and the levels of Collagen 1 A, Collagen 3 A and α-SMA were also greatly increased in the pulmonary interstitial fibroblasts co-cultured with exosomes (*p* *<* 0.05). In conclusion, the proliferation of pulmonary interstitial fibroblasts is stimulated by M2 macrophages through the secretion of exosomes.

### M2 macrophage-secreted exosomes carry miR-328 into pulmonary interstitial fibroblasts

**Fig. 6:**
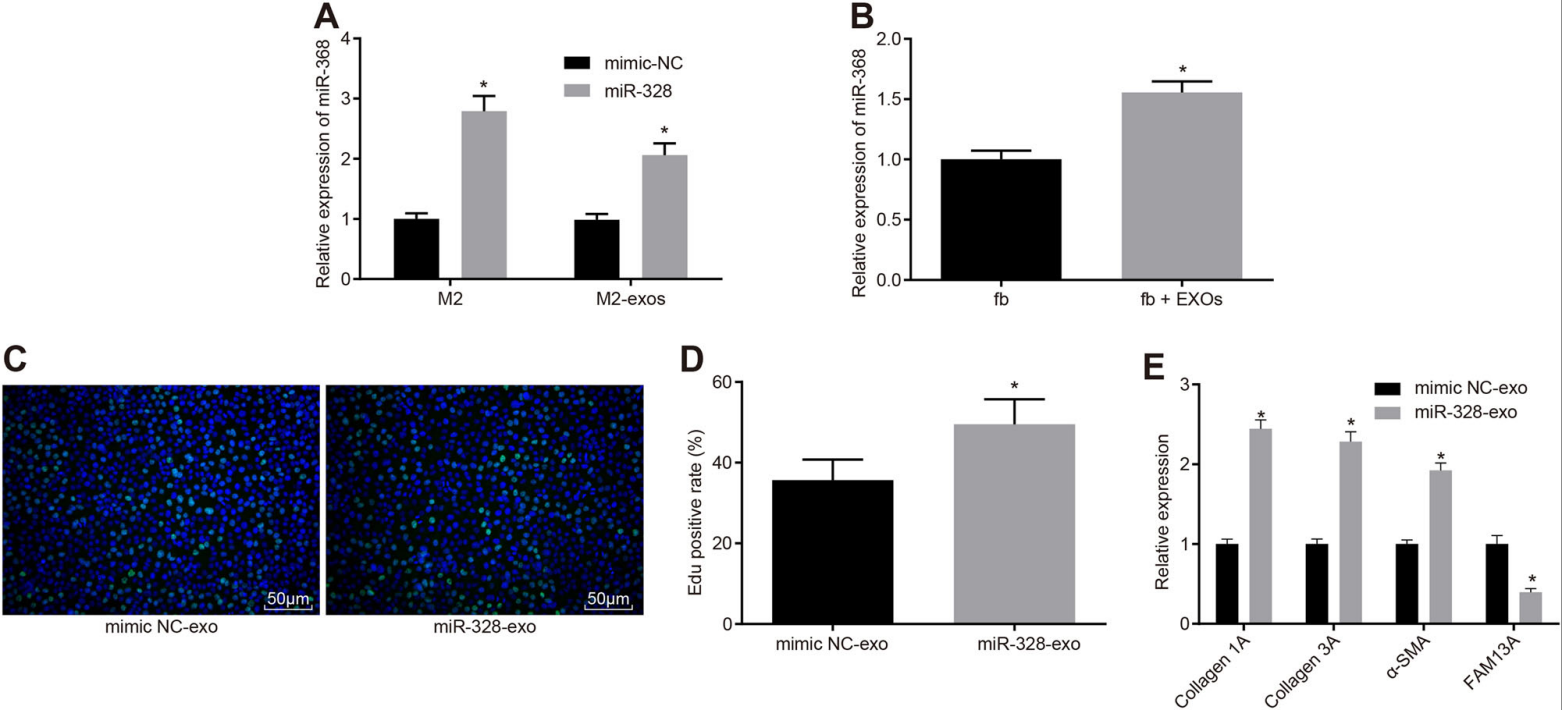
**M2 macrophage-exosomes carry miR-328 into pulmonary interstitial fibroblasts**. **a** miR-328 expression in macrophages and exosomes examined by *RT-qPCR*. **b** miR-328 expression in pulmonary interstitial fibroblasts after a coculture with exosomes, as measured by *RT-qPCR*, * *p* *<* 0.05 *vs*. pulmonary interstitial fibroblasts cocultured with exosomes. **c** EdU labeling to examine the proliferation of pulmonary interstitial fibroblasts in the mimic NC-exo and miR-328-exo groups (200 × ). **d** Quantification of the results in **b**, *, *p* *<* 0.05 *vs*. the mimic NC-exo group; **e** Expression of Collagen 1A, Collagen 3A, α-SMA and FAM13A in the mimic NC-exo and miR-328-exo groups measured by *RT-qPCR*, * *p* *<* 0.05 *vs*. the mimic NC-exo group. miR-328, microRNA-328; NC, negative control; EdU, 5-ethynyl-2′-deoxyuridine; α-SMA, α-smooth muscle actin; *RT-qPCR*, reverse transcription quantitative polymerase chain reaction; NC, negative control; and exo, exosomes. The results were measurement data and were analyzed using an unpaired *t* test. The results are expressed as the mean ± standard deviation. The experiment was conducted in triplicate

Since the effects of miR-328 and exosomes on PF are similar, we then investigated the association between miR-328 and exosomes. Initially, we examined the expression of miR-328 in macrophages and macrophage-derived exosomes from the rats in the control group and the PF group by *RT-qPCR* (Fig. 6a), and the results revealed that miR-328 expression was significantly increased in the macrophages and exosomes of the rats in the PF group compared with those of the rats in the control group. In addition, we determined the effect of exosomes on miR-328 expression in pulmonary interstitial fibroblasts (Fig. 6b). EdU labeling was also conducted to compare the proliferation of pulmonary interstitial fibroblasts between the mimic NC-exosome (exo) and miR-328-exo groups (Fig. 6c, d). In addition, *RT-qPCR* was performed to determine the expression of FAM13A, Collagen 1 A, Collagen 3 A, and α-SMA (Fig. 6e). miR-328 expression was increased in the pulmonary interstitial fibroblasts cocultured with exosomes compared with the pulmonary interstitial fibroblasts cultured alone (*p* *<* 0.05). Compared with the mimic NC-exo group, the miR-328-exo group exhibited significantly stimulated pulmonary interstitial fibroblast proliferation, reduced expression of FAM13A and increased expression of Collagen 1 A, Collagen 3 A, and α-SMA (*p* *<* 0.05). In conclusion, exosomes carried miR-328 into pulmonary interstitial fibroblasts.

### miR-328 inhibition in M2 macrophages represses the progression of PF in vivo by upregulating FAM13A expression

**Fig. 7:**
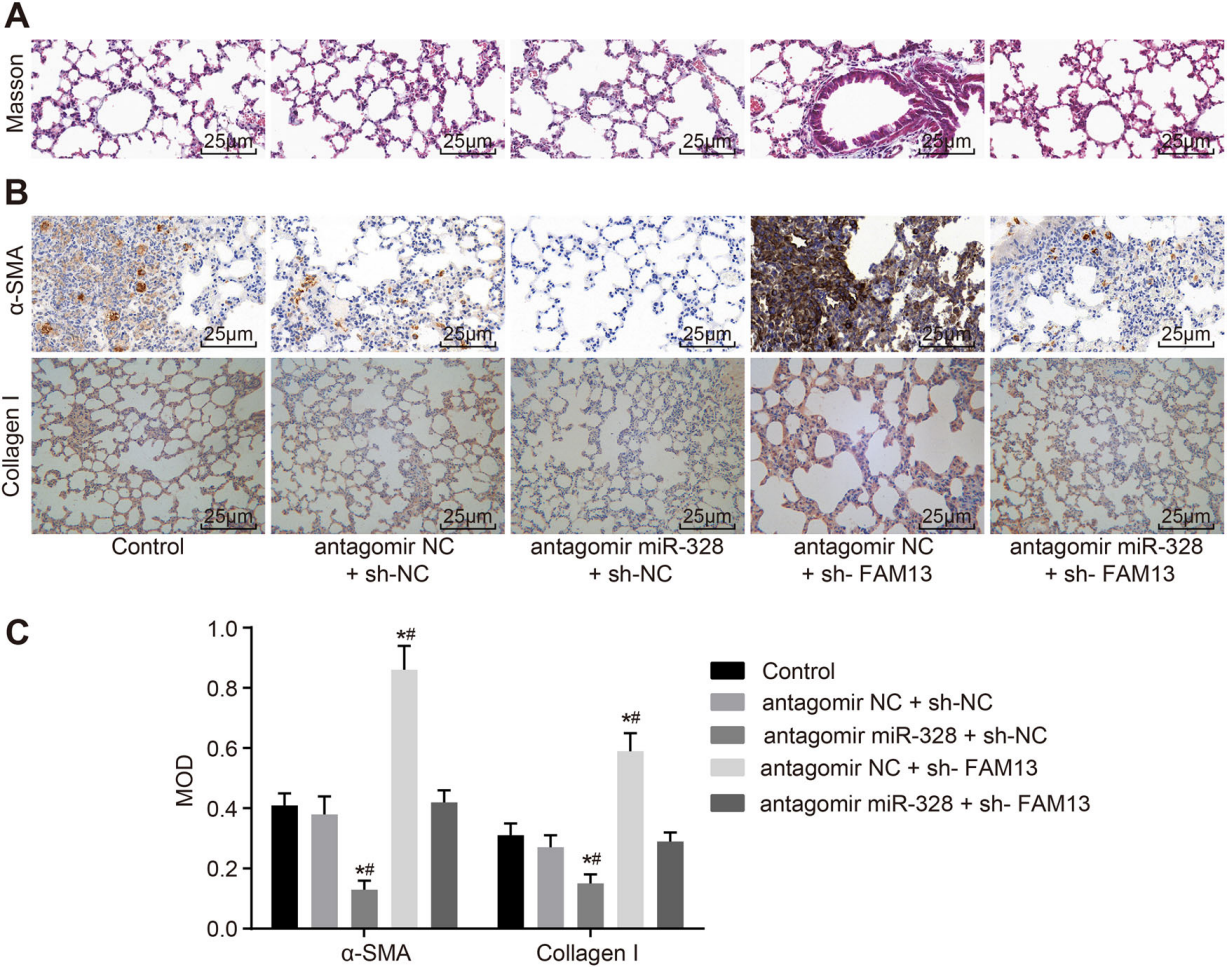
**miR-328 inhibition in M2 macrophages suppresses the progression of PF by targeting FAM13A**. **a** Masson staining to examine the degree of PF in rats after the inhibition of miR-328 and FAM13A (400 × ). **b** IHC to analyze the expression of α-SMA and Collagen I in rats after the inhibition of miR-328 and FAM13A (400 × ). **c** MOD values of α-SMA and Collagen I in rats after the inhibition of miR-328 and FAM13A. miR-328, microRNA-328; PF, pulmonary fibrosis; FAM13A, family with sequence similarity 13, member A; α-SMA, α-smooth muscle actin; IHC, immunohistochemistry; and NC, negative control. * *p* *<* 0.05 *vs*. the control group; # *p* *<* 0.05 *vs*. the antagomir NC + sh-NC group. All results were measurement data and were analyzed using one-way analysis of variance. The results are expressed as the mean ± standard deviation; *n* = 6

Subsequently, we aimed to elucidate whether miR-328 in M2 macrophages is able to promote the pulmonary fibrotic process, so we injected untreated M2 macrophages or M2 macrophages infected with a lentivirus containing antagomir NC + scramble shRNA, antagomir miR-328 + scramble shRNA, antagomir NC + shRNA-FAM13A, or antagomir miR-328 + shRNA-FAM13A into rats in the PF group. Masson staining and IHC were employed to assess the degree of PF and MOD values of α-SMA and Collagen I in each group. As shown in Fig. 7, the degree of fibrosis was significantly decreased, and the MOD values of α-SMA and Collagen I were significantly lower in the antagomir miR-328 + scramble shRNA group compared with the antagomir NC + scramble shRNA group (*p* *<* 0.05). Opposite results were observed in the antagomir NC + shRNA-FAM13A group. Additionally, it is implied that antagomir miR-328 could rescue the worsening of PF caused by shRNA-FAM13A. Taken together, these results indicated that miR-328 inhibition in M2 macrophages suppresses the progression of PF by increasing FAM13A expression.

### Silencing of the exosomal miR-328 of M2 macrophages alleviates the progression of PF in vivo

**Fig. 8:**
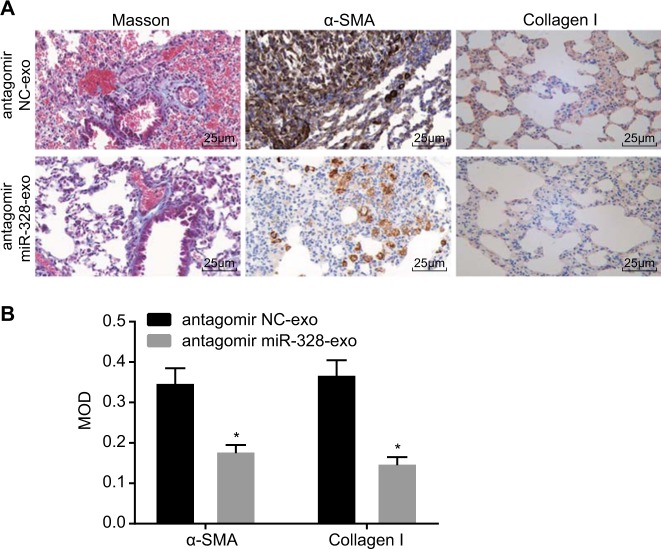
**Knocking down exosomal miR-328 expression in M2 macrophages inhibits the progression of PF in vivo**. **a** The degree of fibrosis and expression of α-SMA and Collagen I observed by Masson staining and IHC, respectively, in the antagomir NC-exo and antagomir miR-328-exo groups (400 × ). **b** MOD values of α-SMA and Collagen I. * *p* *<* 0.05 *vs*. the mimic NC-exo group. miR-328, microRNA-328; NC, negative control; and α-SMA, α-smooth muscle actin. The results were measurement data and were analyzed using an unpaired *t* test. The results are expressed as the mean ± standard deviation. The experiment was conducted in triplicate

To further explore the function of the exosomal miR-328 of M2 macrophages in PF in vivo, we injected exosomes and antagomir NC or antagomir miR-328 into PF rats *via* the tail vein. The degree of PF was observed in the two groups by Masson staining (Fig. 8a), and positive expression of α-SMA and Collagen I was assessed by IHC (Fig. 8a, b).The PF area was inhibited significantly with decreased numbers of α-SMA- and Collagen I-positive cells in the antagomir miR-328-exo group compared with the antagomir NC-exo group (*p* *<* 0.05). Therefore, PF development could be alleviated by silencing exosomal miR-328 derived from M2 macrophages in vivo.

## Discussion

Alveolar macrophages influence the process of PF by initiating an immune response and generating reactive oxygen species^31^. Exosomes are secreted by a variety of cells and participate in intracellular communication and material transportation *via* signaling molecules on the surface of the cell membrane, as well as by modulating cell fusion^32^. Exosomes derived from macrophages can suppress cell migration^33^. There are few studies that have studied the underlying mechanism by which PF is affected by miR, so we conducted this study to explore how exosomal miR-328 secreted by M2 macrophages influences PF. Consequently, we concluded that exosomes derived from M2 macrophages carrying overexpressed miR-328 were able to aggravate PF.

The first intriguing discovery is that exosomes can be secreted by M2 macrophages. Recovery from inflammatory processes and the maintenance of a basal anti-inflammatory environment in tissues are allowed by shifting between proinflammatory (M1) and anti-inflammatory (M2) states of macrophage polarization^34^. The importance of macrophages in atherogenesis is that they help to form foam cells, which results in the production of various proinflammatory mediators^35^. In lung injury, macrophages are recognized as the most abundant inflammatory cells after neutrophils degranulate and play important roles in the development of fibrosis disorders, including PF^36,37^. Of importance, two sources of alveolar macrophages are the circulating monocyte pool and an intermediate lung macrophage pool, and macrophages are alternatively activated in the lungs of patients with PF^38^. Correspondingly, bleomycin-induced PF can be ameliorated by decreasing M2 macrophage production through chop deficiency^37^ and can be promoted by enhancing the M2 phenotype;^39^ therefore, M2 macrophages are useful antifibrotic targets. In our study, we found that exosomes can be derived from M2 macrophages and that M2 macrophage-derived exosomes can carry miR-328 into pulmonary interstitial fibroblasts. Exosomes are vital for substance transportation as well as communication between cells, which also plays an important role in the wound repair function of stem cells^32^. Similar to our paper, a previous study indicated that miR-21-abundant exosomes can be produced by M2 macrophages^40,41^. In addition, macrophage-derived miR-155-enriched exosomes contribute to enhanced fibroblast inflammation in cardiac injury^42^. Hypoxic epithelial ovarian cancer-derived exosomes carry miR-940 to stimulate M2 polarization of macrophages^43^. It has also been revealed that miR-21 is expressed at a high level in exosomes and M2 macrophages in gastric cancer, suggesting that the silencing of miR-21 in M2 macrophage-derived exosomes could inhibit cisplatin resistance in gastric cancer^44^. Previous studies have reported that collagen promotes the expression of the macrophage inflammatory factors CCL18, IL-1ra and CCL2 and that the upregulation of CD204 expression by collagen exposure enhances the development of human PF^45^. Additionally, it has been revealed that the alternative activation of M2 macrophages functions as a causative agent in PF and that the tyrosine phosphatase Shp2 in alveolar macrophages represses the development of M2-associated pulmonary fibrosis^46^.

Moreover, we also detected that miR-328 expression was greatly upregulated during PF progression. Mizuno *et al*. illustrated that IPF development can be aggravated by the aberrant expression of miRs^47^. For instance, miR-3675–3p, miR-21, miR-1229 and miR-155 are four miRs with upregulated expression in IPF^48^. A recent study reported that miR-328, which is regulated by macrophage-derived reactive oxygen species, may provide a novel target in gastrointestinal cancer^49^. Another study proved that miR-328 is overexpressed in infiltrating glioma cells and promotes glioma cell invasion and proliferation^50^. We also found that miR-328 negatively regulated FAM13A expression and that knocking down FAM13A expression could promote the proliferation of pulmonary interstitial fibroblasts. FAM13A has been demonstrated to affect lung function and be involved in some frequently occurring chronic lung diseases such as COPD, pulmonary fibrosis, lung cancer and asthma^51^. Interestingly, a FAM13A polymorphism is recognized as a factor impacting the prognosis of patients with IPF^19^. Moreover, silencing FAM13A is able to promote epithelial cell proliferation by decreasing β-catenin degradation^52^. Hence, these findings are in accordance with our result that FAM13A silencing enhanced the proliferation of pulmonary interstitial fibroblasts.

Furthermore, it is worth noting that M2 macrophage-secreted exosomes and exosomal miR-328 can upregulate the levels of Collagen 1A, Collagen 3A and α-SMA through downregulating FAM13A expression. Reduced apoptosis and enhanced proliferation in human lung fibroblasts have been reported to be related to increased collagen production induced by oncostatin M^53^. Knocking down miR-133a and miR-29b expression has an impact on the myocardial fibrosis caused by Ang II-dependent hypertension through regulating the expression of collagen 1A1^54^. α-SMA is a marker of pulmonary fibroblast proliferation and differentiation, with TGF-1 acting as a key factor mediating lung fibrosis^55^. A previous study found that the combination of TGF-β and siFAM13A could increase the level of α-SMA^56^.

**Fig. 9:**
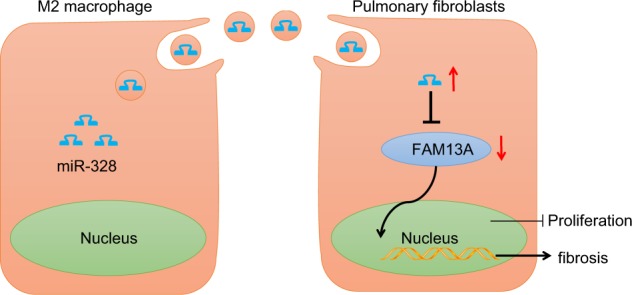
Exosomal miR-328 in M2 macrophages regulates PF progression via FAM13A. miR-328 exhibited upregulated expression in rats with PF and was carried by M2 macrophage-derived exosomes. M2 macrophage-derived exosomes with silenced miR-328 conferred resistance to pulmonary interstitial fibroblast proliferation and inhibited PF by directly upregulating FAM13A expression

In conclusion, our study demonstrated that miR-328-containing exosomes derived from M2 macrophages stimulate pulmonary fibrosis via silencing FAM13A in a rat model (Fig. 9). It should be noted that the sample size in the human experiments was insufficient, indicating that further human experiments are required. Although miR-based therapeutics are still in their infancy, our findings revealed that miR-328 can be regarded as a potential treatment target in PF in the future.

## References

[1] Li LC, Kan LD (2017). Traditional Chinese medicine for pulmonary fibrosis therapy: Progress and future prospects. J. Ethnopharmacol..

[2] Wynn TA (2011). Integrating mechanisms of pulmonary fibrosis. J. Exp. Med..

[3] Fernandez IE, Eickelberg O (2012). New cellular and molecular mechanisms of lung injury and fibrosis in idiopathic pulmonary fibrosis. Lancet.

[4] Raghu G (2011). An official ATS/ERS/JRS/ALAT statement: idiopathic pulmonary fibrosis: evidence-based guidelines for diagnosis and management. Am. J. Respir. Crit. Care. Med..

[5] Oh CK, Murray LA, Molfino NA (2012). Smoking and idiopathic pulmonary fibrosis. Pulm. Med..

[6] Xu Y (2012). Clinical characteristics of patients with lung cancer and idiopathic pulmonary fibrosis in China. Thorac. Cancer.

[7] Rathinasabapathy A (2016). Therapeutic potential of adipose stem cell-derived conditioned medium against pulmonary hypertension and lung fibrosis. Br. J. Pharmacol..

[8] Roszer T (2015). Understanding the Mysterious M2 Macrophage through Activation Markers and Effector Mechanisms. Mediat. Inflamm..

[9] Misharin AV (2017). Monocyte-derived alveolar macrophages drive lung fibrosis and persist in the lung over the life span. J. Exp. Med..

[10] Bardi GT, Smith MA, Hood JL (2018). Melanoma exosomes promote mixed M1 and M2 macrophage polarization. Cytokine.

[11] Witwer, K. W. et al. Standardization of sample collection, isolation and analysis methods in extracellular vesicle research. *J. Extracell. Vesicles* **2**, (2013). 10.3402/jev.v2i0.20360.eCollection2013. 10.3402/jev.v2i0.20360 PMC3760646 24009894

[12] Marote A, Teixeira FG, Mendes-Pinheiro B, Salgado AJ (2016). MSCs-Derived exosomes: cell-secreted nanovesicles with regenerative potential. Front. Pharmacol..

[13] Dejima H, Iinuma H, Kanaoka R, Matsutani N, Kawamura M (2017). Exosomal microRNA in plasma as a non-invasive biomarker for the recurrence of non-small cell lung cancer. Oncol. Lett..

[14] Arora S (2011). MicroRNA-328 is associated with (non-small) cell lung cancer (NSCLC) brain metastasis and mediates NSCLC migration. Int J. Cancer.

[15] Lin CH, Chiang MC, Chen YJ (2018). MicroRNA-328 inhibits migration and epithelial-mesenchymal transition by targeting CD44 in nasopharyngeal carcinoma cells. Onco. Targets Ther..

[16] Artlett CM, Sassi-Gaha S, Hope JL, Feghali-Bostwick CA, Katsikis PD (2017). Mir-155 is overexpressed in systemic sclerosis fibroblasts and is required for NLRP3 inflammasome-mediated collagen synthesis during fibrosis. Arthritis Res. Ther..

[17] Jiang Z (2016). A chronic obstructive pulmonary disease susceptibility gene, FAM13A, regulates protein stability of beta-Catenin. Am. J. Respir. Crit. Care. Med..

[18] Hobbs BD (2017). Genetic loci associated with chronic obstructive pulmonary disease overlap with loci for lung function and pulmonary fibrosis. Nat. Genet..

[19] Hirano C (2017). FAM13A polymorphism as a prognostic factor in patients with idiopathic pulmonary fibrosis. Respir. Med..

[20] Shannon P (2003). Cytoscape: a software environment for integrated models of biomolecular interaction networks. Genome Res..

[21] Sheng W (2013). Cooperation among Numb, MDM2 and p53 in the development and progression of pancreatic cancer. Cell Tissue Res..

[22] Odegaard JI (2007). Macrophage-specific PPARgamma controls alternative activation and improves insulin resistance. Nature.

[23] Lumeng CN, Bodzin JL, Saltiel AR (2007). Obesity induces a phenotypic switch in adipose tissue macrophage polarization. J. Clin. Invest..

[24] Huang M (2002). IL-7 inhibits fibroblast TGF-beta production and signaling in pulmonary fibrosis. J. Clin. Invest..

[25] Tachibana K (2011). Polymyxin-B hemoperfusion for acute exacerbation of idiopathic pulmonary fibrosis: serum IL-7 as a prognostic marker. Sarcoidosis Vasc. Diffus. Lung. Dis..

[26] Dweep H, Gretz N (2015). miRWalk2.0: a comprehensive atlas of microRNA-target interactions. Nat. Methods.

[27] Zhao D (2018). Cardiomyocyte derived miR-328 promotes cardiac fibrosis by paracrinely regulating adjacent fibroblasts. Cell. Physiol. Biochem..

[28] Du W (2016). MicroRNA-328, a potential anti-fibrotic target in cardiac interstitial fibrosis. Cell. Physiol. Biochem..

[29] Philip K (2017). HIF1A up-regulates the ADORA2B receptor on alternatively activated macrophages and contributes to pulmonary fibrosis. FASEB J..

[30] Xiang J (2016). Neotuberostemonine attenuates bleomycin-induced pulmonary fibrosis by suppressing the recruitment and activation of macrophages. Int. Immunopharmacol..

[31] Larson-Casey JL, Deshane JS, Ryan AJ, Thannickal VJ, Carter AB (2016). Macrophage Akt1 kinase-mediated mitophagy modulates apoptosis resistance and pulmonary fibrosis. Immunity.

[32] Li MY, Liu DW, Mao YG (2017). Advances in the research of effects of exosomes derived from stem cells on wound repair. Zhonghua Shao Shang Za Zhi.

[33] Lee HD, Kim YH, Kim DS (2014). Exosomes derived from human macrophages suppress endothelial cell migration by controlling integrin trafficking. Eur. J. Immunol..

[34] Sierra-Filardi E, Vega MA, Sanchez-Mateos P, Corbi AL, Puig-Kroger A (2010). Heme Oxygenase-1 expression in M-CSF-polarized M2 macrophages contributes to LPS-induced IL-10 release. Immunobiology.

[35] van Tits LJ (2011). Oxidized LDL enhances pro-inflammatory responses of alternatively activated M2 macrophages: a crucial role for Kruppel-like factor 2. Atherosclerosis.

[36] Yao Y (2016). Chop deficiency protects mice against bleomycin-induced pulmonary fibrosis by attenuating M2 macrophage production. Mol. Ther..

[37] Wynn TA, Barron L (2010). Macrophages: master regulators of inflammation and fibrosis. Semin. Liver. Dis..

[38] Murray LA (2010). Serum amyloid P therapeutically attenuates murine bleomycin-induced pulmonary fibrosis via its effects on macrophages. PLoS ONE.

[39] Ayaub EA (2016). GRP78 and CHOP modulate macrophage apoptosis and the development of bleomycin-induced pulmonary fibrosis. J. Pathol..

[40] Hsieh CH, Tai SK, Yang MH (2018). Snail-overexpressing Cancer Cells Promote M2-Like Polarization of Tumor-Associated Macrophages by Delivering MiR-21-Abundant Exosomes. Neoplasia.

[41] Zhao H (2018). Exosomes from adipose-derived stem cells attenuate adipose inflammation and obesity through polarizing M2 Macrophages and Beiging in White Adipose Tissue. Diabetes.

[42] Wang C (2017). Macrophage-derived MIR-155-containing exosomes suppress fibroblast proliferation and promote fibroblast inflammation during cardiac injury. Mol. Ther..

[43] Chen X (2017). Exosomes derived from hypoxic epithelial ovarian cancer deliver microRNA-940 to induce macrophage M2 polarization. Oncol. Rep..

[44] Zheng P (2017). Exosomal transfer of tumor-associated macrophage-derived miR-21 confers cisplatin resistance in gastric cancer cells. J. Exp. Clin. Cancer Res..

[45] Stahl M (2013). Lung collagens perpetuate pulmonary fibrosis via CD204 and M2 macrophage activation. PLoS ONE.

[46] Tao B (2014). Myeloid-specific disruption of tyrosine phosphatase Shp2 promotes alternative activation of macrophages and predisposes mice to pulmonary fibrosis. J. Immunol..

[47] Mizuno K (2017). MicroRNAs in non-small cell lung cancer and idiopathic pulmonary fibrosis. J. Hum. Genet..

[48] Li P (2014). Expression analysis of serum microRNAs in idiopathic pulmonary fibrosis. Int. J. Mol. Med..

[49] Ishimoto T (2014). Macrophage-derived reactive oxygen species suppress miR-328 targeting CD44 in cancer cells and promote redox adaptation. Carcinogenesis.

[50] Delic S (2014). MiR-328 promotes glioma cell invasion via SFRP1-dependent Wnt-signaling activation. Neuro. Oncol..

[51] Jin Z (2015). Regulation of nuclear-cytoplasmic shuttling and function of Family with sequence similarity 13, member A (Fam13a), by B56-containing PP2As and Akt. Mol. Biol. Cell.

[52] Brandsma CA, Timens W (2016). The translation from risk allele to biological function in chronic obstructive pulmonary disease. What’s in it for FAM13A?. Am. J. Respir. Crit. Care. Med..

[53] Scaffidi AK (2002). Oncostatin M stimulates proliferation, induces collagen production and inhibits apoptosis of human lung fibroblasts. Br. J. Pharmacol..

[54] Castoldi G (2012). MiR-133a regulates collagen 1A1: potential role of miR-133a in myocardial fibrosis in angiotensin II-dependent hypertension. J. Cell. Physiol..

[55] Liu G (2010). miR-21 mediates fibrogenic activation of pulmonary fibroblasts and lung fibrosis. J. Exp. Med..

[56] Corvol H (2018). FAM13A is a modifier gene of cystic fibrosis lung phenotype regulating rhoa activity, actin cytoskeleton dynamics and epithelial-mesenchymal transition. J. Cyst. Fibros..

